# Epidemiology of Digital Dermatitis in Western Canadian Feedlot Cattle

**DOI:** 10.3390/ani14071040

**Published:** 2024-03-29

**Authors:** Sarah Erickson, Calvin Booker, Jiming Song, Eugene Janzen, Murray Jelinski, Karen Schwartzkopf-Genswein

**Affiliations:** 1 TELUS Agriculture and Consumer Goods, Okotoks, AB T1S 2A2, Canada; sarah.erickson@telusagcg.com (S.E.); calvin.booker@telusagcg.com (C.B.); jasmine.song@telusagcg.com (J.S.); eugene.janzen@telusagcg.com (E.J.); 2 Faculty of Veterinary Medicine, University of Calgary, Calgary, AB T2N 4Z6, Canada; 3 Western College of Veterinary Medicine, University of Saskatchewan, Saskatoon, SK S7N 5B4, Canada; 4 Lethbridge Research and Development Centre, Agriculture and Agri-Food Canada, Lethbridge, AB T1J 4B1, Canada; karen.genswein@agr.gc.ca

**Keywords:** ruminant lameness, epidemiology and modelling, digital dermatitis

## Abstract

**Simple Summary:**

The knowledge base surrounding digital dermatitis (DD) primarily relies on research pertaining to the dairy industry, little of which can be extrapolated to DD development in feedlot cattle. This study represents the largest retrospective study, to date, on the diagnosis of DD in western Canadian feedlot cattle. Utilizing records from western Canadian feedlots this study revealed associations between sex, placement quarter, and acquisition source, and DD at the animal-level, alongside an association between feedlot population size and the presence or absence of diagnosed DD cases at the feedlot level. These findings will drive future research related to understanding the development and transmission of DD in feedlot cattle as it pertains to these risk factors.

**Abstract:**

Digital dermatitis (DD) is an emerging disease in feedlot cattle. Our objective was to identify animal- and feedlot-level risk factors for DD by analyzing individual animal health records (*n* = 1,209,883) and feedlot-level records from western Canadian feedlots (*n* = 28) between 2014 and 2018, inclusive. The risk of a DD diagnosis was higher (incidence rate ratio (IRR) = 2.08, 95% CI 1.52 to 2.86) in cattle sourced from confined background operations (CB) versus cattle sourced from auction markets (AM). Conversely, ranch direct (RD) cattle were (IRR = 0.02, 95% CI 0.04 to 0.30) lower risk than AM cattle of being diagnosed with DD. The risk of being diagnosed with DD was higher in females than in males. The magnitude of the risk in females over males was influenced by annual DD incidence in low morbidity years (2014, 2017, and 2018) (IRR = 2.02, 95% CI 1.27 to 3.19), medium morbidity years (2016) (IRR = 2.95, 95% CI 1.64 to 5.33), and high morbidity years (2015) (IRR = 5.41, 95% CI 3.27 to 8.95). At the feedlot-level, the risk of a diagnosis of DD was lower in small capacity (SCF) versus large capacity feedlots (LCF) (IRR = 0.24, 95% CI 0.05 to 0.76). Future research should focus on identifying factors that may propagate disease transmission between cattle of different sexes and from different acquisition sources.

## 1. Introduction

Digital dermatitis (DD) is a multifactorial, polymicrobial [1] contagious [2], hoof-related lameness (HRL), ubiquitous in the dairy industry world-wide [1,3,4], and of growing concern in the beef industry [5]. While the pathogenesis of DD is not fully understood, it is widely accepted that phylotypes of the genus *Treponema* are associated with the development of this disease [2,3,6,7]. In the USA dairy industry, *T. denticola*, *T. phagedenis* [1,8,9], *T. pallidum*, *T. vincentii* [8], *T. medium* [1,9], *T. maltophilum*, and *T. putidum* [9] have been identified as the most common *Treponema* spp. isolated from DD lesions, with *T. phagedenis* being identified in all DD lesion stages [1]. Research on beef cattle in the United Kingdom corroborates these results [10,11], providing evidence that DD presents similarly in dairy cattle and beef cattle, supporting treponemes as the primary etiologic agent of DD regardless of cattle type [11,12]. 

Clinical DD is characterized by ulcerative, circular lesions, circumscribed by raised hyperkeratotic skin, commonly involving the skin proximal to the interdigital cleft and the plantar aspect of the hind feet [3,5]. Active DD lesions present as a distinctive strawberry-red coloration, while chronic DD lesions commonly have hypertrophied hairs (papilliform projections) surrounding the lesion borders and epithelial growths (parakeratotic hyperkeratosis) [3]. Histologically, DD lesions are characterized by a loss of stratum corneum, epidermal hyperplasia, and reactive inflammation [3,5,6]. Rasumussen et al. [13] proposed that non-infectious etiologies lead to skin defects that allow for the secondary invasion of spirochetes. More recently, Pirkkalainen et al. [14] conducted a histological study of DD lesions and found that spirochetes only existed in areas of tissue necrosis. They posited that thickening of the epidermis results in ischemic necrosis of the dermal papillae, allowing for the secondary invasion of spirochetes. 

Digital dermatitis has been identified in 4% (29/739) of culled adult beef cattle in the southeastern USA [15]. More recently, research on western Canadian beef cattle has estimated the cumulative incidence of DD to range from 2.5% (71/2854), in cattle from two finishing feedlots monitored from November 2018 to November 2019 [16], to 6% (894/14,900) in cattle from two finishing feedlots from 2016 to 2018 [16]. It is estimated that DD contributes to between 9.21% [17], 17.66% [16], and 25.5% [18] of lameness diagnoses in western Canadian feedlot cattle. A Northern USA feedlot study determined that the average days on feed (DOFs) at DD diagnosis was 104 DOFs [19], indicating that DD is a lameness more commonly identified later in the feeding period. 

The western Canadian beef cattle industry is generally segregated into three sectors: calf production, backgrounding, and finishing. Beef (cow-calf) producers supply the industry with calves that are born in the spring and weaned in the fall of the year. Most calves are then sold into feedlots via auctions or directly (ranch direct), where they are placed on high-caloric rations and finished (fattened) for approximately 8 months before they are sold for slaughter. Alternatively, following weaning, they may be placed in confined backgrounding operations where they are fed lower caloric rations to gradually grow in weight and skeletal structure. Depending on market conditions, the cattle are backgrounded for a number of months, or in some instances retained, and placed on pasture (grass cattle) and then sold to the feedlots in the fall as yearlings. The finishing feedlots are considered intensive livestock operations with cattle raised in earthen-floor pens enclosed by wooden porosity pens. A typical pen would hold approximately 300 cattle, but it varies by feedlot. Cattle are fed a caloric-dense total mixed ration (TMR) that is conducive to supporting maximal weight gain and carcass quality. Bovine respiratory disease is the most common cause of morbidity and mortality in western Canadian feedlot cattle, followed by lameness, particularly foot rot [18]. However, digital dermatitis is becoming increasingly more common in beef cattle [20]. 

There is a paucity of research pertaining to the epidemiology of DD in beef cattle. In the dairy industry, the main risk factors for DD are poor pen conditions, specifically cattle hygiene, moisture, and depth of mud, slurry, and manure in pens [21,22,23]. In agreement with this, one western Canadian feedlot study found that pens with average mud depths of 6 cm to >20 cm increased the odds of cattle developing DD from 8.45 to 13.9 times relative to pens with average mud depths of <6 cm [19]. Factors affecting pen conditions, specifically manure and environmental slurry, have also been implicated in the environmental persistency of DD pathogens [4,23,24]. A western Canadian study identified multiple risk factors associated with the development of lameness in feedlot cattle: body weight, cattle source, pen density, percentage of forage in the diet, season, cattle type, and sex [18,25]. However, specific investigation into the association of these, and other risk factors, with the development of DD has yet to be performed. Due to the endemic nature of DD in dairy operations [1,22], placement of dairy cattle in feedlots is speculated as a means for the introduction of DD in feedlots [26]; however, this has not been confirmed. 

The objectives of this study were two-fold: (1) describe the epidemiology as well as the animal- and feedlot-level risk factors associated with the diagnosis of DD in feedlot cattle, and (2) provide recommendations for future research regarding the prevention and control of DD in feedlot cattle.

## 2. Materials and Methods

Data were acquired from 28 western Canadian feedlots subscribed to the *i*FHMS Consolidated Database (Feedlot Health Management Services, a division of TELUS Agriculture Solutions Inc. (FHMS), Irricana, AB, Canada). For the feedlot-level analysis, twenty-seven of the feedlots in this study were in Alberta, Canada, and one feedlot was in Saskatchewan, Canada. Animal placement data for this study spanned the period of 1 January 2014 to 31 December 2018.

In 2010, FHMS consultants educated feedlot personnel on diagnosing and treating hoof-related lameness (HRL), including DD. The following scenario is typical of most large commercial feedlots. Pen checkers surveilling the pens on foot or on horseback would make a presumptive diagnosis of lameness based on the animal’s locomotion, specifically weight bearing, head bobbing, stride length, and an arched back [27]. Lame cattle are then ‘pulled’ from the pen and moved to a treatment facility within the feedlot. Here the treatment crew provides a more in-depth inspection of the affected limb. However, unlike the dairy industry, most commercial feedlot facilities lack the time and resources to closely examine every hoof. Thus, a putative DD diagnosis would be based on finding lesions consistent with any stage of the disease along with the lack of another lesion (foot rot or toe tip necrosis) that would account for the lameness. Staging lesions and performing routine follow-ups was not performed because the primary goal is to identify the cause of lameness and provide the most appropriate treatment. In the current study, treatment crews would have also consulted with the feedlot veterinarians, when available, to arrive at a correct diagnosis. The feedlot veterinarians were also consulted in the event of a DD outbreak. 

### 2.1. Animal-Level Data Analysis

Two separate statistical models were used. The first model was used to identify animal-level risk factors for identifying lame cattle with DD lesions. The second model identified the feedlot-level risk factors for identifying cases of DD in lame animals. Animal-level factors were queried from iFHMS utilizing Microsoft^®^ Office Access 365 ProPlus (Microsoft Corporation, Redmond, WA, USA). These factors included feedlot identification (ID); animal ID; production lot number (≥head/lot); placement date; placement year; age class (calf/yearling); breed (beef/dairy); sex (heifer, steer, or bull); acquisition source (auction market (AM), confined backgrounding operation (CB), grass cattle (GC), ranch direct (RD), or unknown); DD treatment record; treatment date; and days on feed (DOFs) at time of treatment. All DD treatment data were restricted to the first diagnosis and first day of treatment; relapses were not included in the analysis. The time period of interest for which a DD diagnosis could have been made corresponded to an individual animal’s duration of time in the feedlot, referred to hereafter as the ‘feeding period’. Once queried, the data were imported into a Microsoft^®^ Office Excel 365 ProPlus spreadsheet (Microsoft Corporation, Redmond, WA, USA) via the Power Pivot add-in and manipulated using data analysis expressions to create the final dataset of risk factors for DD: placement year, age class, sex, placement quarter, acquisition source, feedlot population size, and breed category. Age class was defined as calf or yearling, based on age at the time of arrival to the feedlot. Sex combined steers and bulls together as “males” and heifers as “females”. Placement quarter was defined based on the calendar year: quarter 1 (Qtr1) from January to March, quarter 1 (Qtr2) from April to June, quarter 3 (Qtr3) from July to September, and quarter 4 (Qtr4) from October to December. Placement quarter was used as a categorical variable to describe trends in cattle placement volumes and weather fluctuations throughout the calendar year. Acquisition sources included AM, CB, GC, and RD. Cattle with unknown sources were removed from the dataset. Feedlot population size was defined as small capacity feedlots (SCF) (<10,000 cattle) versus large capacity feedlots (LCF) (≥10,000 cattle). This variable was created using the weighted average of placements in each feedlot between 2014 and 2018. Breed category was defined as beef breeds versus dairy breeds, in which dairy breeds included any beef on dairy cross-cattle. Animal-level DD treatment status (yes/no) and treatment (Tx) date were included. 

Of the 28 feedlots included in the study, 14 had one or more diagnosed cases of DD between 2014 and 2018. For the individual animal-level analysis, only data pertaining to cattle from those 14 feedlots were considered. The GENMOD procedure in SAS^®^ Version 9.4 (SAS Institute Inc., Cary, NC, USA) was used to conduct univariable and multivariable Poisson regression using a log linear model to identify individual animal-level risk factors associated with the diagnosis of DD, adjusted for hierarchical clustering of observations (production lot nested within Feedlot ID) with generalized estimating equations (GEE). The outcome for the Poisson regression models was the incidence risk of DD (DD%). Variables were screened in univariable models and those with *p* ≤ 0.20 were included in the multivariable Poisson model. A new variable (Annual DD-Level) representing the incidence of DD by year(s) was created: LowMorb (≤0.29%; years 2014, 2017, and 2018), MediumMorb (0.30% to 0.39%; year 2016), and HighMorb (≥0.40%, year 2015). A second variable was created to group the incidence of DD by the placement period (quarter) in which cattle entered the feedlot (DD-Placement Period): Qtr1 (January to March), Qtr2/Qtr3 (April to June and July to September), and Qtr4 (October to December).

The final model was selected for goodness-of-fit via the lowest quasi-likelihood under the independence model criterion (QIC). Collinearity was assessed utilizing Cramer’s V as a measure of effect for the Pearson’s chi-square test of independence, in conjunction with the descriptive statistics for each variable (Appendix A; Table A1). Based on an inclusion level of *p* < 0.20 at the univariable level (Appendix A; Table A2), a multivariable model was constructed by sequentially including variables with the largest effect parameters using a forward selection process. A multivariable model was also constructed using backward selection to rule out any discrepancies in the biological significance of the included fixed effects. In both cases, variables remained in the multivariable model based on *p* < 0.05 and the risk factors assessed based on the defined univariable inclusion level were Annual DD-Level, DD-Placement Period, acquisition source, sex, age class, breed category and feedlot population size. The final model was assessed for two-way and three-way interactions of fixed effects using pair-wise comparisons. Only significant interactions were reported. Incidence rate ratios (IRR) of DD per animal per feeding period, 95% confidence intervals (CI), and the associated *p*-values were reported for the Poisson regression.

### 2.2. Feedlot-Level Data Analysis

The categorical feedlot-level risk factors included bedding type, feedlot program type (continuous or seasonal placements), history of placing dairy breeds (yes/no), and feedlot type (backgrounding or finishing). Data on the proportion of cattle placement types including auction market sourced, backgrounding operation sourced, grass cattle, ranch direct sourced, beef, dairy, calf, yearling, female, and male, and within each quarter of the calendar year (quarter (Qtr) 1 through 4) were organized into four quartiles. Quartile 0 represented 0 to 25%, quartile 1 represented 26 to 50%, quartile 3 represented 51 to 75%, and quartile 4 represented 76 to 100%. Information on temperature, humidity, and precipitation were collected using the GPS coordinates of each feedlot to locate the nearest weather station via the Alberta Climate Information Service website (Government of Alberta, 2020) for feedlots located in Alberta and the Environment Canada website (Government of Canada, 2020) for the feedlot located in Saskatchewan. Feedlot GPS coordinates were mapped in Google Earth (Google Landsat/Copernicus Data SIO, NOAA, U.S. Navy, NGA, GEBCO, 2020) for the analysis of elevation and geographic location. All feedlot-level variables, and additional descriptions, are detailed in Table 1. 

Feedlot program was categorized as continuous, where cattle were placed throughout the year, with no period where the feedlot was empty, and seasonal, where cattle were only placed at certain times of the year and there were periods where the feedlot had low inventory or was empty. Geographic location was categorized as south or north, where the feedlot’s location demarcation was the city of Airdrie, Alberta. From daily weather observations, averages over the placement period (2014 to 2018) were calculated for humidity, temperature, and precipitation for the fall and winter season (Qtr1/Qtr4) and the spring and summer season (Qtr2/Qtr3). 

The GENMOD procedure in SAS^®^ Version 9.4 (SAS Institute Inc., Cary, NC, USA) was used to conduct univariable Poisson regression in a log linear model to identify feedlot-level risk factors associated with the development of digital dermatitis (DD). The outcome for the Poisson regression model was case versus control. Variables were screened in the univariable model to identify effect levels and *p*-values for the multivariable model building; a cut-off of *p* ≤ 0.20 was chosen for inclusion in the multivariable model. 

The final model was selected for goodness-of-fit via the lowest Akaike information criterion (AIC). The final variables chosen for inclusion were population size, feedlot program, geographic location, fall/winter humidity average, and spring/summer temperature average. Forward selection was then used with variables having the largest effect parameters included sequentially in the forward selection process. Backward selection was attempted, to test for biologically significant differences and confounding; however, the model would not converge. Incidence rate ratios (IRR), 95% CIs, and the associated *p*-values were reported for the Poisson regression.

## 3. Results and Discussion

### 3.1. Epidemiology of Digital Dermatitis

The 14 case feedlots placed 1,209,883 cattle between 1 January 2014 and 31 December 2018. Annual placements ranged from 2692 to 37,266 (average of 17,284) cattle/feedlot/year. Table 2 is the proportion (%) of cattle placements within the different animal-level risk factors by placement year (2014 to 2018).

It must be appreciated that many cases of DD are subclinical and hence do not result in overt lameness [28]. In this study, the pen checkers only pulled animals showing lameness, and there was no effort made to examine non-lame animals for DD lesions. Thus, the epidemiology of DD described herein is more accurately termed the epidemiology of DD in lame feedlot cattle in western Canadian feedlots. This distinction is not unique to our study; rather, the following two studies by Cortes et al. [16,17] both describe the incidence of DD being diagnosed in lame animals, which does make the datasets comparable. In our study, the average annual incidence risk of DD was 0.29%, with a range of 0.21% to 0.43%. Whereas Cortes et al. [17] reported that a three-year study (2016 to 2018) of two feedlots found the incidence of DD diagnoses to be 1.16% (894/77,115). The lower incidence in our study may be explained in part by the inclusion of DD relapses by Cortes et al. [17]. The second study by Cortes et al. [16] reported a cumulative incidence of 2.5% (71/2854) or an incidence proportion of 2.1 DD cases per 1000 cattle per month for cattle from two finishing feedlots studied from 2018 to 2019. It is unclear if relapses were included in that study. In the present study, the incidence proportion of DD was 0.05 DD cases per 1000 cattle per month. At the lot level for the DD case feedlots, DD was diagnosed in 461/3667 production lots, with the average incidence risk in these lots being 1.84% (range: 0.04% to 32.00%). 

**Figure 1:**
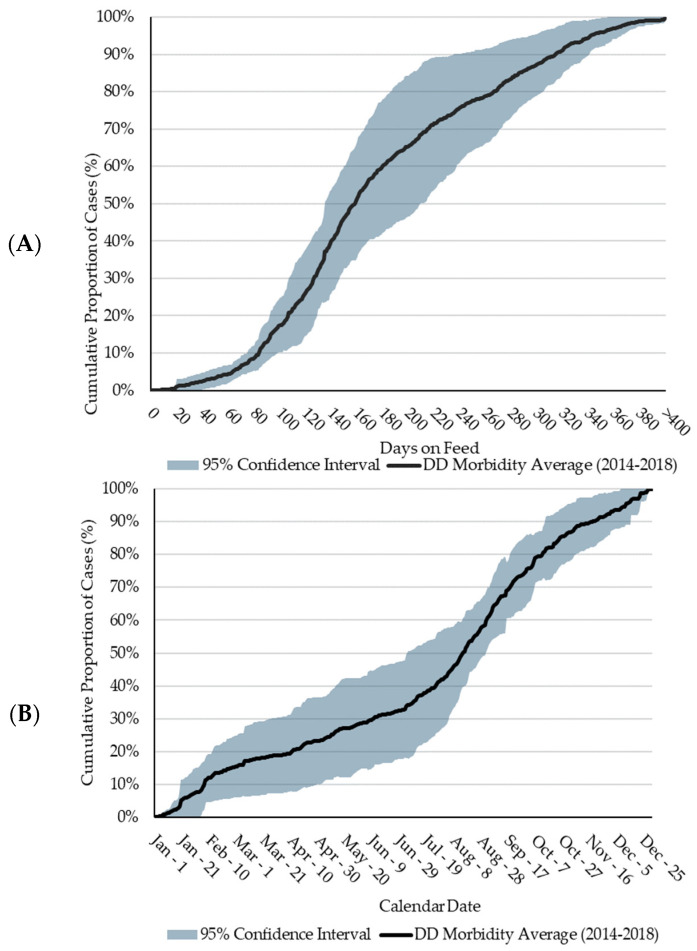
Five-year average (2014 to 2018) of the cumulative proportional distribution of digital dermatitis (DD) cases. Shading represents the 95% confidence interval (CI). (**A**): Distribution by days on feed (DOFs). (**B**): Distribution by calendar date.

The cumulative distribution of DD cases between 2014 and 2018 follows a nearly identical trend from 0 to 83 DOFs (Figure 1), as illustrated by the narrow 95% CI about the 5-year average during this time. Eighty percent of DD cases occurred between 83 and 314 DOFs (median = 158 DOFs; average = 168 DOFs). This is higher than a US feedlot study which determined the mean DOF at the time of DD diagnosis to be 104 DOFs [19]. In general, DD cases follow a sigmoidal trend by DOF, with most cases occurring at a later DOF. Variation in the 95% CI (Figure 1) increases substantially between 140 and 305 DOFs due to the distribution of DD cases in 2015 reaching 90% by 197 DOFs, which was over 100 days earlier than the other four years. Further investigation identified a group of yearlings placed in a LCF which largely contributed to the incidence of DD in 2015. Due to heavier arrival weights, relative to calves, yearlings typically do not remain on feed past 200 days. This factor, alongside the high proportion of DD in yearlings in 2015, confounded the shape of the DD distribution curve. At the feedlot level, it is unlikely that 2015 differed significantly from other years.

Less than 30% of DD cases occurred between late-November and early-April, a timeframe of approximately 110 days. Referring to the DOF observations, less than 30% of cases occurred prior to 126 DOFs. These two observations may be attributed to the seasonal nature of the western Canadian feedlot industry, wherein most cattle are placed in the fall and early winter months and would not reach > 126 DOFs until later in the spring of the following year. As the feeding period continues, few DD cases are observed until July when DD cases rapidly increased, a time frame which, for fall-placed feedlot cattle, coincides with a later DOF. This timeframe is consistent with conclusions from Cortes et al. [16] who reported a difference (*p* < 0.05) in the number of DD cases during the summer versus the other seasons, with 49.2% of cases being diagnosed in the summer. Conceivably, heat stress could be a factor related to the increase in DD over the summer. However, the higher incidence continues into cooler fall months. 

The largest variation in DD reporting occurred from early-March to mid-August, where the variation increased to approximately 30%. This variation may be a function of weather fluctuations and consequential declines in pen conditions. The increase in DD cases observed from July to mid-November is consistent with the increase in DD cases at a later DOF for cattle placed in the fall of the previous year. Furthermore, from early-August and onwards, the 95% CI about the five-year average narrows significantly, suggesting consistency in case increases in DD during these months regardless of placement year or feedlot. Seasonality, in terms of placement patterns and weather fluctuations, plays a significant role in DD case occurrence. However, the complexity of this association, and the role of a later DOF in DD lesion development requires further research.

### 3.2. Individual Animal-Level Analysis Results

The multivariable model and interaction evaluations are summarized in Table 3 for the individual animal-level analysis.

There was a significant two-way interaction between sex and Annual DD-Level. Females displayed a consistently higher risk of being diagnosed with DD than their male counterparts, and the magnitude of the risk in females over males was affected by the incidence risk of DD within different placement years (Annual DD-Level categories). Females had a 2.02 (95% CI 1.27 to 3.19, *p =* 0.003), 2.95 (95% CI 1.64 to 5.33, *p* < 0.001), and 5.41 (95% CI 3.27 to 8.95, *p* < 0.001) times higher incidence rate ratio than males in LowMorb years, MediumMorb years, and HighMorb years, respectively. This corresponds to a higher risk for DD diagnosis in females over males that increases in magnitude based on higher annual DD incidence. In contrast, a southeastern USA slaughterhouse study of culled adult beef cattle found a significant difference in the development of DD lesions in male versus female cattle (*p =* 0.017) [15]. In that study, however, all males were intact adults, making it difficult to compare to the current study. A western Canadian study evaluating the association of gender with the outcome of foot rot (FR), leg/hoof injury (INJ), joint infections (JI), and lameness with no visible swelling (LNVS) reported that the odds of developing LNVS was 2.46 times higher in steers than in heifers [25]. From their definition, LNVS may have included DD; however, a direct comparison to the DD analysis in the current study cannot be made. The present study represents the first in western Canada to evaluate sex as a risk factor for DD, specifically. 

Interestingly, the later DOF trend of DD cases and the higher risk of diagnosing DD cases in females is comparable to the epidemiology of atypical interstitial pneumonia (AIP) in feedlot cattle, wherein AIP affects females at a higher rate than males [29]. Similar to the DOF range for DD diagnoses, the average DOF at the time of death for cattle diagnosed with AIP ranges from 114 to 136 days [29]. For both AIP and DD, it is unclear what is driving the difference between females and males. One theory may be differing levels of estrogen and testosterone in males and females [30]. This dimorphism increases male susceptibility to gastrointestinal and respiratory bacterial diseases and increases female susceptibility to urinary tract bacterial infections [30]. Future research regarding this potential association with bacterial infections of the hoof is needed. Another theory concerning management differences between males and females is the feeding of melengestrol acetate (MGA) to heifers. Previous research has reported decreased rates of AIP in heifers where MGA was removed from the diet [29,31]. Feeding MGA has yet to be researched for any associations with developing DD. Another potential research avenue would be to assess whether there is any correlation between AIP and DD diagnosis, particularly in late-DOF heifers.

Poor pen conditions, particularly mud depth in feedlot pens [16], is associated with DD development. In the current study, cattle placed in Qtr1 and Qtr2/Qtr3 were at a higher risk of being diagnosed with DD compared to cattle placed in Qtr4, IRR = 1.65 (95% CI 1.06 to 2.58, *p =* 0.027) and IRR = 2.24 (95% CI 1.48 to 3.39, *p* < 0.001), respectively. As previously stated, over 80% of DD cases in this study occurred between 83 and 314 DOFs, and between the months of mid-April and mid-November, with the most observed from mid-July to the end of September. As time progresses from mid-April to mid-November, cattle placed in Qtr1 and Qtr2/Qtr3 would approach or exceed between 100 and 300 DOFs. It is speculated that the later DOF of Qtr1 and Qtr2/Qtr3 placements over the spring and summer seasons, alongside fluctuations in weather and pen conditions common to western Canada during that time, influence the risk of DD development in Qtr1 and Qtr2/Qtr3 placements relative to cattle placed in Qtr4. 

Cattle sourced from CB were at higher risk for being diagnosed with DD than cattle sourced from AM (IRR = 2.08, 95% CI 1.52 to 2.86, *p* < 0.001). Conversely, cattle sourced from RD were at lower risk than cattle sourced from AM (IRR = 0.11, 95% CI 0.04 to 0.30, *p* < 0.001). Studies have determined that stress, associated with the commingling of unrelated cattle, handling, and transportation to and from cattle systems, all play a significant role in disease susceptibility, pathogen load, and morbidity [32,33]. Cattle from CB operations and GC are unique from cattle from other sources in that the handling, transportation, and commingling of unrelated cattle may occur frequently and long-term, particularly if cattle were purchased from auctions. Those factors arise again when CB and GC cattle are purchased and placed in a feedlot. Such commingling increases the likelihood that novel diseases, such as DD, may be introduced into the population. Auctions act as a short-term consignment step between other cattle systems, mainly marketing cattle from RD sources [34], reducing the time available for commingling and disease transfer. In contrast to other cattle sources, particularly CB and GC, it is less likely that RD sourced cattle have been commingled with unrelated cattle prior to feedlot arrival, reducing the potential for DD introduction and transmission.

Breed category was not significant in this analysis. However, it has been speculated that the placement of dairy breeds in feedlots introduces DD to the population [26]. This speculation is born from the knowledge that DD is endemic in dairy operations and cattle in dairies have likely been exposed to DD [1,3,4]. An important factor that may be overlooked here is the utilization of calf ranches as an intermediary step between dairy operations and feedlots. Typically, calf ranches receive day-old calves from dairies and background them to an appropriate weight prior to marketing them to finishing feedlot yards. Due to their movement into a calf ranch directly following birth, it can be speculated that those day-old calves do not actually get exposed to DD in the dairy operation. If that speculation is true, those dairy calves would not necessarily introduce DD into feedlot populations. Future research should continue to investigate the potential association between breed and DD development, while accounting for potential differences between dairy cattle acquired from dairies and those acquired from calf ranches who would have spent little to no time physically at the dairy.

Age class was also not significant in this analysis. This is particularly interesting as multiple western Canadian studies have documented significant differences between calves and yearlings and the outcome of becoming lame [18,25,35]. Each of these studies included multiple diseases under their definition of lameness but none evaluated DD as a singular entity. This gives insight for future research that although age class plays an important role in development lameness, this may not hold true when evaluating DD by itself. This also highlights the importance of analyzing diseases individually to better understand their contributions to the overall impact of lameness in feedlot cattle.

Considering the animal-level results, three main research areas are recommended. Firstly, focus on identifying risk factors within different acquisition sources that may predispose cattle to DD infection in a feedlot setting. Secondly, further study into why heifers were at higher risk than steers and bulls. Lastly, reevaluate the potential association between breed category (beef versus dairy) and the risk of DD development. Although breed category was not a significant risk factor in this study, it is often speculated that placement of dairy cattle in feedlots may introduce DD to the population, propagating its transmission to beef cattle. Future research is needed to validate these speculations.

### 3.3. Feedlot-Level Analysis Results

**Figure 2:**
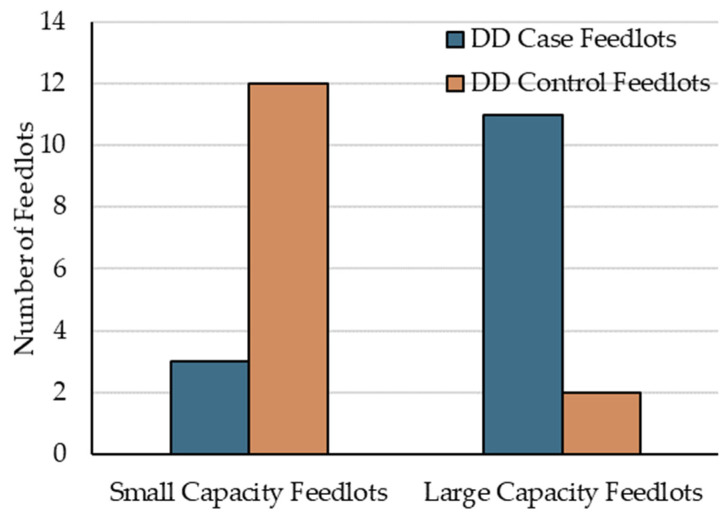
Distribution of case and control feedlots by population size, defined as small capacity feedlots (SCF) and large capacity feedlots (LCF).

At the feedlot-level, the only significant finding was that SCF were at lower risk of having diagnosed DD cases than LCF (IRR = 0.24, 95% CI 0.05 to 0.76, *p =* 0.027). Interestingly, in addition to the current research coinciding with Woolums’ [23] report of higher AIP risk in females, at the feedlot-level, AIP was also more likely (*p* < 0.01) to occur in larger (≥10,000 cattle placed annually) versus smaller feedlots (<10,000 cattle placed annually) [29,36]. Figure 2 illustrates the distribution of population size within case and control feedlots. 

Relative to feedlot capacity, it is suspected that LCF are more likely than SCF to acquire cattle from multiple sources, multiple times per year. Resultant increases in variability in cattle health and disease susceptibility, particularly as it relates to stressors including handling, transportation, and commingling, may propagate the increased risk for DD in LCF [32,33]. Despite its role as a risk factor for DD, research evaluating pen slurry as a primary infection reservoir and/or route of transmission for DD found that treponemes associated with the development of DD were only detected in slurry samples obtained from dairy herds with reported DD problems [4]. This finding indicates that DD treponemes are not naturally present in pen environments and, therefore, cannot be the primary infection reservoir for DD-free herds [4]. This suggests that higher variability in cattle placements and sources increases the chances of introducing novel diseases, such as DD, into the feedlot. With fewer cattle placed annually, SCF may be less likely to have an animal introduce DD into the yard, particularly if SCF acquire cattle from the same sources each year. If DD pathogens are introduced into pen environments, slurry and feces can be significant vectors in DD transmission [4,23].

The variable feedlot program (seasonal versus continuous) was excluded from the statistical model, as no DD case feedlots had a seasonal feedlot program. However, the finding that none of the seasonal feedlots had DD cases suggests that an all-in/all-out strategy of feeding cattle may be a factor in controlling DD. Within a seasonal feeding program, there are periods during the year in which the entire feedlot is empty. Given that poor pen conditions, with increased mud and slurry, are a risk factor for DD [3,16,21], it is speculated that continuous feeding programs may be more conductive to creating poor pen conditions than the seasonal feeding programs. Furthermore, a United Kingdom dairy study found that environmental temperatures above 45 °C resulted in nonviable DD treponemes, suggesting that *Treponema* spp. would not survive composting or pasteurization techniques [37]. Perhaps warmer summer months contribute to the death of treponemes during the empty pen periods of seasonal feeding programs. It is also of note that all the seasonal feedlots were also SCF, suggesting a confounding relationship between the reduced DD risk in SCF and their utilization of seasonal feeding programs. Alternatively, it may indicate that the results observed here were only attributed to feedlot size.

Future research should focus on feedlot size and type (continuous vs. seasonal) as these factors may give insight into interactions with other variables and potential mitigation strategies. While the size of the feedlot itself is relatively static, other factors that differ between SCF and LCF may yield important insights into the presence or absence of DD, such as cattle acquisition sources and cattle types. Additionally, future research into seasonal and continuous feedlot programs, particularly within different sizes of feedlots, and their potential association with the presence or absence of DD is an important area of focus. This may also involve evaluating different acquisition sources and different cattle types within seasonal feedlots.

A limitation of this study is that the 28 feedlots included in this study may not represent the western Canadian feedlot industry. However, this is the largest and broadest study of DD in western Canada to date. Secondly, all DD lesions were diagnosed by feedlot personnel or FHMS veterinarians; however, these diagnoses may or may not have included lifting the feet for examination. This may have resulted in misdiagnosis and misclassification, which could have impacted result outcomes and represents a second limitation in this study. Thirdly, only lame cattle were examined for DD lesions, and, hence, the true incidence of DD may be much higher than reported. 

## 4. Conclusions

At the feedlot level, the risk of having lame cattle with DD lesions was higher in the LCF versus SCF. The feedlot program (seasonal vs. continuous) may be of biological significance, albeit not statistical, as there was an absence of DD case feedlots utilizing seasonal placement patterns.

At the animal-level, the risk of diagnosing DD was higher in females than males. In years with high DD morbidity, the risk in females becomes even greater than their male counterparts. Considering acquisition source, the risk of DD cases was lowest in the ranch direct cattle and highest in cattle sourced from confined backgrounding operations. The risk of developing DD was highest in cattle placed in late-summer and early-fall (Qtr2/Qtr3) relative to cattle placed in Qtr4. This is no doubt confounded by when cattle typically enter feedlots in western Canada. Age class, breed category, and population size were not risk factors for identifying lame cattle with DD. 

## Figures and Tables

**Table 1:** Feedlot-level risk factors and their corresponding categories collected from 28 western Canadian feedlots. Categories represent the variable type that was utilized in statistical modelling. Risk factor description provides additional context to each specific risk factor and how they were defined.

Feedlot Risk Factor	Categories	Risk Factor Description
Auction-Placed Quartiles	Quartiles (0, 1, 2, 3)	Auction cattle
Background-Placed Quartiles	Quartiles (0, 1, 2, 3)	Confined backgrounded cattle
Bedding Type	Straw/Shavings/Both	Feedlot pen bedding type
Beef-Placed Quartiles	Quartiles (0, 1, 2, 3)	Beef breeds
Calf-Placed Quartiles	Quartiles (0, 1, 2, 3)	Calf-age cattle placed
Dairy-Placed Quartiles	Quartiles (0, 1, 2, 3)	Dairy or dairy-cross breeds
Elevation Quartiles	Quartiles (0, 1, 2, 3)	Elevation above sea-level
Fall/Winter Humidity Avg.	Upper 50%/Lower 50%	January–March and October–December
Fall/Winter Precipitation Avg.	Upper 50%/Lower 50%	January–March and October–December
Fall/Winter Temperature Avg.	Upper 50%/Lower 50%	January–March and October–December
Feedlot Program	Continuous/Seasonal	Method of cattle stocking
Feedlot Type	Finishing/Backgrounding	Method of cattle feeding
Female-Placed Quartiles	Quartiles (0, 1, 2, 3)	Female cattle (heifers)
Geographic Location	North/South	Reference: Airdrie, Alberta
Grass-Placed Quartiles	Quartiles (0, 1, 2, 3)	Grass cattle
History of Dairy Breeds	Yes/No	Dairy or dairy cross-breeds
Male-Placed Quartiles	Quartiles (0, 1, 2, 3)	Male cattle (steers and bulls)
Population Size	Small/Large Capacity	Avg. placements (2014–2018)
Qtr1-Placed Quartiles	Quartiles (0, 1, 2, 3)	Qtr1 (January–March) Placements
Qtr2-Placed Quartiles	Quartiles (0, 1, 2, 3)	Qtr2 (April–June) Placements
Qtr3-Placed Quartiles	Quartiles (0, 1, 2, 3)	Qtr3 (July–September) Placements
Qr4-Placed Quartiles	Quartiles (0, 1, 2, 3)	Qtr4 (October–December) Placements
Ranch Direct-Placed Quartiles	Quartiles (0, 1, 2, 3)	Ranch direct cattle
Spring/Summer Humidity Avg.	Upper 50%/Lower 50%	April–June and July–September
Spring/Summer Precipitation Avg.	Upper 50%/Lower 50%	April–June and July–September
Spring/Summer Temperature Avg.	Upper 50%/Lower 50%	April–June and July–September
Yearling-Placed Quartiles	Quartiles (0, 1, 2, 3)	Yearling-age cattle placed

**Table 2:** Proportion (% and number) of cattle placed by year and the combined 5-year average for animal-level risk factors. The percentages associated with each risk factor sums to 100% as does the placements across the 5-year study period.

Animal-Level Risk Factor	2014	2015	2016	2017	2018	Average
Placement Quarter (Qtr)						
Qtr 1 (January to March)	23.87% (54,785)	19.34% (42,354)	23.08% (53,463)	22.61% (59,684)	18.39% (48,880)	21.46% (51,833)
Qtr 2 (April to June)	18.13% (41,600)	14.81% (32,435)	17.02% (39,426)	16.47% (43,475)	18.54% (49,284)	16.99% (41,244)
Qtr 3 (July to September)	13.35% (30,635)	21.69% (47,493)	16.21% (37,537)	18.87% (49,821)	25.50% (67,800)	19.12% (46,657)
Qtr 4 (October to December)	44.66% (102,494)	44.15% (96,659)	43.69% (101,198)	42.05% (110,984)	37.57% (99,876)	42.42% (102,242)
Acquisition Source						
Auction Market	62.31% (143,000)	64.22% (140,596)	60.13% (139,269)	66.22% (174,785)	62.39% (165,865)	63.05% (152,703)
Confined Backgrounding	23.23% (53,307)	20.84% (45,617)	22.87% (52,964)	21.84% (57,662)	24.48% (65,066)	22.65% (54,923)
Grass	11.55% (26,510)	12.17% (26,642)	14.60% (33,806)	10.25% (27,051)	7.09% (18,861)	11.13% (26,574)
Ranch Direct	2.92% (6697)	2.78% (6086)	2.41% (5585)	1.69% (4466)	6.04% (16,048)	3.17% (7776)
Population Size						
Small Capacity Feedlots	8.57% (19,678)	8.46% (18,529)	5.85% (13,552)	7.09% (18,710)	6.11% (16,253)	7.22% (17,344)
Large Capacity Feedlots	91.43% (209,836)	91.54% (200,412)	94.15% (218,072)	92.91% (245,254)	93.89% (249,587)	92.78% (224,632)
Age Class						
Calves	45.76% (105,037)	48.55% (106,293)	48.14% (111,495)	50.96% (134,514)	49.01% (130,279)	48.48% (117,524)
Yearlings	54.24% (124,477)	51.45% (112,648)	51.86% (120,129)	49.04% (129,450)	50.99% (135,561)	51.52% (124,453)
Sex						
Female (heifers)	36.02% (82,668)	36.35% (79,588)	37.51% (86,882)	32.86% (86,744)	28.64% (76,139)	34.28% (82,404)
Male (steers and bulls)	63.98% (146,846)	63.65% (139,353)	62.49% (144,742)	67.14% (177,220)	71.36% (189,701)	65.72% (159,572)
Breed Category						
Beef Breeds	92.90% (213,222)	92.56% (202,646)	93.12% (215,698)	93.30% (246,269)	82.64% (219,691)	90.90% (219,505)
Dairy Breeds	7.10% (16,292)	7.44% (16,295)	6.88% (15,926)	6.70% (17,695)	17.36% (46,149)	9.10% (22,471)

**Table 3:** Results of multivariable statistical modelling for associations between animal-level risk factors and developing digital dermatitis. The incidence rate ratio (IRR) is the measure of association and statistical significance was determined at *p* < 0.05.

Animal Level Risk Factor	Pop% ^1^	DD% ^2^	IRR	*p*-Value	Estimate	IRR 95% CI ^3^
Sex; Annual DD-Level						
HighMorb-Female	6.58	0.91	5.41	<0.001	1.688	3.27 to 8.95
HighMorb-Male	11.52	0.16	-	-	-	-
MediumMorb-Female	7.18	0.57	2.95	<0.001	1.083	1.64 to 5.33
MediumMorb-Male	11.96	0.20	-	-	-	-
LowMorb-Female	20.30	0.33	2.02	0.003	0.702	1.27 to 3.19
LowMorb-Male	42.46	0.17	-	-	-	-
DD-Placement Period						
Qtr1	21.42	0.27	1.65	0.027	0.503	1.06 to 2.58
Qtr2/Qtr3	36.33	0.44	2.24	0.000	0.807	1.48 to 3.39
Qtr4	42.25	0.15	-	-	-	-
Acquisition Source						
Confined Background (CB)	22.70	0.48	2.08	<0.001	0.734	1.52 to 2.86
Grass Cattle (GC)	10.98	0.38	1.65	0.060	0.502	0.98 to 2.79
Ranch Direct (RD)	3.21	0.02	0.11	<0.001	−2.207	0.04 to 0.30
Auction Market (AM)	63.11	0.20	-	-	-	-

^1^ Proportional percent of the population for a given animal-level risk factor accounts. ^2^ Incidence risk of digital dermatitis (DD) within the population of a given animal-level risk factor. ^3^ Confidence interval (CI) shown as incidence rate ratio (IRR) values.

## Data Availability

Data are contained within the article and Supplementary Materials.

## Supplementary Materials

The following supporting information can be downloaded at Master of Science thesis: The epidemiology of hoof-related lameness in western Canadian feedlot cattle, https://harvest.usask.ca/server/api/core/bitstreams/e0439db2-ab0d-44cc-a36a-09e70eafef15/content, accessed on 28 September 2023.

## Appendix A

**Table A1:** Descriptive statistics on population numbers and digital dermatitis (DD) morbidity for animal-level risk factors in 14 western Canadian feedlots.

Animal-Level Risk Factor	Pop Mean	Pop Median	Pop Range	DD% Mean	DD% Median	DD% Range
Annual DD-Level						
HighMorb (2015)	15,639	14,433	5033 to 30,593	0.35%	0.01%	0.00% to 2.46%
MediumMorb (2016)	16,545	17,360	3296 to 32,396	0.36%	0.04%	0.00% to 1.90%
LowMorb (2014, 2017, 2018)	18,079	16,180	2692 to 37,266	0.19%	0.00%	0.00% to 1.47%
DD-Placement Period						
Q1 (January–March)	18,512	19,547	2162 to 36,162	0.31%	0.03%	0.00% to 1.86%
Q2/Q3 (April–September)	15,697	16,183	0 to 34,692	0.37%	0.07%	0.00% to 1.86%
Q4 (October–December)	36,515	26,240	14,229 to 84,036	0.16%	0.06%	0.00% to 0.58%
Acquisition Source						
Auction Market	54,537	38,991	0 to 148,016	0.23%	0.08%	0.00% to 0.89%
Confined Backgrounding	19,615	7332	0 to 82,188	0.47%	0.13%	0.00% to 3.03%
Grass	9491	238	0 to 44,448	0.18%	0.00%	0.00% to 0.91%
Ranch Direct	2777	1360	0 to 18,603	0.05%	0.00%	0.00% to 0.50%
Population Size						
Small Capacity Feedlots	28,907	29,161	24,330 to 33,231	0.17%	0.16%	0.00% to 0.34%
Large Capacity Feedlots	102,106	98,287	49,262 to 164,179	0.29%	0.28%	0.00% to 1.01%
Age Class						
Calves	41,973	36,553	1479 to 105,844	0.16%	0.00%	0.00% to 0.80%
Yearlings	44,448	48,910	0 to 103,536	0.29%	0.24%	0.00% to 1.03%
Sex						
Female (heifers)	29,430	24,807	1 to 83,043	0.35%	0.15%	0.00% to 1.23%
Male (steers and bulls)	56,990	66,537	7993 to 117,637	0.19%	0.16%	0.00% to 0.63%
Breed Category						
Beef Breeds	78,395	75,100	0 to 164,179	0.24%	0.10%	0.00% to 1.01%
Dairy Breeds	8026	0	0 to 82,188	0.04%	0.00%	0.00% to 0.41%

**Table A2:** Univariate statistical analysis of animal-level risk factors and the incidence rate ratio (IRR) of developing digital dermatitis (DD).

Animal-Level Risk Factor	IRR	*p*-Value	Estimate	95% CI
Placement Year				
2014	1.08	0.813	0.074	0.59 to 1.98
2015	2.03	0.005	0.706	1.24 to 3.30
2016	1.59	0.080	0.465	0.95 to 2.68
2017	1.12	0.659	0.117	0.67 to 1.89
2018	-	-	-	-
Placement Quarter				
Quarter 1 (Qtr1)	1.87	0.004	0.625	1.22 to 2.87
Quarter 2 (Qtr2)	3.17	<0.001	1.153	2.04 to 4.92
Quarter 3 (Qtr3)	2.66	<0.001	0.980	1.65 to 4.29
Quarter 4 (Qtr3)	-	-	-	-
Acquisition Source				
Confined Background (CB)	2.39	<0.001	0.873	1.74 to 3.30
Grass Cattle (GC)	1.92	0.016	0.652	1.13 to 3.25
Ranch Direct (RD)	0.11	<0.001	−2.232	0.04 to 0.29
Auction Market (AM)	-	-	-	-
Population Size				
Small Capacity Feedlots (SCF)	0.57	0.363	−0.561	0.17 to 1.91
Large Capacity Feedlots (LCF)	-	-	-	-
Age Class				
Calves	0.53	<0.001	−0.638	0.39 to 0.72
Yearlings	-	-	-	-
Sex				
Female (heifers)	2.85	<0.001	1.049	2.08 to 3.91
Male (steers and bulls)	-	-	-	-
Breed Category (Reference: Dairy)				
Beef Breeds	0.90	0.526	−0.105	0.65 to 1.25
Dairy Breeds	-	-	-	-

**Table A3:** Range (minimum to maximum) and average temperature (temp.) in degrees Celsius (°C), percent humidity (%), and millimeters (mm) of precipitation observations by year and quarter (Qtr.).

Time Period	Air Temp. Range (°C)	Air Temp. Average (°C)	Humidity Range (%)	Humidity Average (%)	Precipitation Range (mm)	Precipitation Average (mm)
2014						
Qtr1	−41.00 to 15.70	−13.01	18.20 to 101.10	75.46	0.00 to 113.90	11.86
Qtr2	−24.20 to 32.22	8.97	10.70 to 103.90	66.33	0.00 to 327.30	47.20
Qtr3	−8.20 to 35.60	15.87	14.40 to 102.30	70.05	0.00 to 480.70	103.50
Qtr4	−40.60 to 26.60	−3.03	14.80 to 103.40	76.58	0.00 to 601.90	130.03
2015						
Qtr1	−42.30 to 22.50	−7.71	10.80 to 103.70	76.46	0.00 to 715.80	149.99
Qtr2	−11.50 to 35.60	11.13	6.20 to 102.30	55.08	0.00 to 929.20	184.28
Qtr3	−8.60 to 37.90	15.87	0.30 to 103.00	66.23	0.00 to 1082.60	239.56
Qtr4	−30.20 to 27.10	−0.97	1.00 to 103.00	75.41	0.00 to 1203.80	266.09
2016						
Qtr1	−35.40 to 19.80	−4.25	4.80 to 103.00	75.89	0.00 to 1318.30	286.04
Qtr2	−12.22 to 33.70	11.81	5.30 to 104.00	57.58	0.00 to 1531.70	321.51
Qtr3	−5.60 to 33.10	15.46	13.20 to 104.10	68.90	0.00 to 1685.10	375.76
Qtr4	−35.00 to 23.40	−2.49	10.50 to 105.00	77.02	0.00 to 1806.30	402.29
2017						
Qtr1	−37.00 to 19.10	−7.87	15.90 to 104.60	75.80	0.00 to 1920.20	426.37
Qtr2	−13.60 to 33.33	10.97	11.00 to 103.90	58.08	0.00 to 2133.60	458.59
Qtr3	−7.60 to 37.22	16.88	8.10 to 104.40	56.43	0.00 to 2287.00	511.82
Qtr4	−39.80 to 26.20	−4.10	7.00 to 104.70	72.20	0.00 to 2408.20	542.40
2018						
Qtr1	−41.30 to 15.60	−11.59	16.00 to 103.70	76.92	0.00 to 2522.10	564.50
Qtr2	−29.40 to 34.50	10.53	9.00 to 104.70	58.17	0.00 to 2735.50	600.19
Qtr3	−9.80 to 40.56	14.71	7.10 to 103.10	62.90	0.00 to 2888.90	662.72
Qtr4	−32.00 to 24.90	−3.11	8.50 to 100.00	76.21	0.00 to 3010.10	679.48

## References

[1] Krull A.C., Shearer J.K., Gorden P.J., Cooper V.C., Phillips G.J., Plummer P.J. (2014). Deep sequencing analysis reveals temporal microbiota changes associated with development of bovine digital dermatitis. Infect. Immun..

[2] Gomez A., Cook N.B., Bernardoni N.D., Rieman J., Dusick A.F., Hartshorn R., Socha M.T., Read D.H., Döpfer D. (2014). An experimental infection model to induce digital dermatitis infection in cattle. J. Dairy Sci..

[3] Read D.H., Walker R.L. (1998). Papillomatous digital dermatitis (footwarts) in California dairy cattle: Clinical and gross pathological findings. J. Vet. Diagn. Investig..

[4] Klitgaard K., Strube M.L., Isbrand A., Jensen T.K., Nielsen M.W., Dudley E.G. (2017). Microbiota analysis of an environmental slurry and its potential role as a reservoir of bovine digital dermatitis pathogens. Appl. Environ. Microbiol..

[5] Wilson-Welder J.H., Alt D.P., Nally J.E. (2015). The etiology of digital dermatitis in ruminants: Recent perspectives. Vet. Res..

[6] Walker R.L., Read D.H., Loretz K.J., Nordhausen R.W. (1995). Spirochetes isolated from dairy cattle with papillomatous digital dermatitis and interdigital dermatitis. Vet. Microbiol..

[7] Stamm L.V., Bergen H.L., Walker R.L. (2002). Molecular typing of papillomatous digital dermatitis-associated Treponema isolates based on analysis of 16S-23S ribosomal DNA intergenic spacer regions. J. Clin. Microbiol..

[8] Zinicola M., Higgins H., Lima S., Machado V., Guard C., Bicalho R. (2015). Shotgun meta-genomic sequencing reveals functional genes and microbiome associated with bovine digital dermatitis. PLoS ONE.

[9] Zinicola M., Lima F., Lima S., Machado V., Gomez M., Döpfer D., Guard C., Bicalho R. (2015). Altered microbiomes in bovine digital dermatitis lesions, and the gut as a pathogen reservoir. PLoS ONE.

[10] Sullivan L.E., Carter S.D., Blowey R., Duncan J.S., Grove-White D., Evans N.J. (2013). Digital dermatitis in beef cattle. Vet. Rec..

[11] Sullivan L.E., Evans N.J., Blowey R.W., Grove-White D.H., Clegg S.R., Duncan J.S., Carter S.D. (2015). A molecular epidemiology of treponemes in beef cattle digital dermatitis lesions and comparative analyses with sheep contagious ovine digital dermatitis and dairy cattle digital dermatitis lesions. Vet. Microbiol..

[12] Plummer P.J., Krull A. (2017). Clinical Perspectives of Digital Dermatitis in Dairy and Beef Cattle. Vet. Clin. N. Am. Food. Anim. Pract..

[13] Rasmussen M., Capion N., Klitgaard K., Rogdo T., Fjeldaas T., Boye M., Jensen T.K. (2012). Bovine digital dermatitis: Possible pathogenic consortium consisting of Dichelobacter nodosus and multiple Treponema species. Vet. Microbiol..

[14] Pirkkalainen H., Riihimäki A., Lienemann T., Anttila M., Kujala-Wirth M., Rajala-Schultz P., Simojoki H., Soveri T., Orro T. (2024). Local and Systemic Inflammation in Finnish Dairy Cows with Digital Dermatitis. Animals.

[15] Brown C.C., Kilgo P.D., Jacobsen K.L. (2000). Prevalence of papillomatous digital dermatitis among culled adult cattle in the southeastern United States. Am. J. Vet. Res..

[16] Cortes J.A., Thomas A., Hendrick S., Janzen E., Pajor E.A., Orsel K. (2021). Risk factors of digital dermatitis in feedlot cattle. Trans. Anim. Sci..

[17] Cortes J.A., Hendrick S., Janzen E., Pajor E.A., Orsel K. (2021). Economic impact of digital dermatitis, foot rot, and bovine respiratory disease in feedlot cattle. Trans. Anim. Sci..

[18] Marti S., Jelinski M.D., Janzen E.D., Jelinski M.J., Dorin C.L., Orsel K., Pajor E.A., Shearer J., Millman S.T., Schwartzkopf-Genswein K.S. (2021). A prospective longitudinal study of risk factors associated with cattle lameness in southern Alberta feedlots. Can. J. Anim. Sci..

[19] Terrell S.P., Reinhardt C.D., Larson C.K., Vahl C.I., Thomson D.U. (2017). Incidence of lameness and association of cause and severity of lameness on the outcome for cattle on six commercial beef feedlots. J. Am. Vet. Med. Assoc..

[20] Kulow M., Merkatoris P., Anklam K.S., Rieman J., Larson C., Branine M., Dopfer D. (2017). Evaluation of the prevalence of digital dermatitis and the effects on performance in beef feedlot cattle under organic trace mineral supplementation. J. Anim. Sci..

[21] Rodríguez-Lainz A., Hird D.W., Carpenter T.E., Read D.H. (1996). Case-control study of papillomatous digital dermatitis in Southern California dairy farms. Prev. Vet. Med..

[22] Relun A., Lehebel A., Bruggink M., Bareille N., Guatteo R. (2013). Estimation of the relative impact of treatment and herd management practices on prevention of digital dermatitis in French dairy herds. Prev. Vet. Med..

[23] Wells S.J., Garber L.P., Wagner B.A. (1999). Papillomatous digital dermatitis and associated risk factors in US dairy herds. Prev. Vet. Med..

[24] Staton G.J., Clegg S.R., Ainsworth S., Armstrong S., Carter S.D., Radford A.D., Darby A., Wastling J., Hall N., Evans N.J. (2021). Dissecting the molecular diversity and commonality of bovine and human treponemes identifies key survival and adhesion mechanisms. PLoS Path..

[25] Davis-Unger J., Schwartzkopf-Genswein K., Pajor E.A., Hendrick S., Marti S., Dorin C., Orsel K. (2019). Prevalence and lameness-associated risk factors in Alberta feedlot cattle. Trans. Anim. Sci..

[26] Hendrick S., Abeysekara S. (2014). The Epidemiology and Treatment Costs of Lameness in Western Canadian Feedlot Cattle.

[27] Larson C., Tomlinson D., Branine M., Mulling C., Dopher D., Edwards T. (2014). Cattle Lameness: Identification, Prevention and Control of Claw Lesions. Zinpro Corporation International Bovine Lameness Committee. Zinpro Corporation. https://www.zinpro.com/resource-center/video/cattle-lameness-identification-prevention-and-control-of-claw-lesions-an-important-book-for-the-beef-and-dairy-industry/.

[28] Thomas A.D., Orsel K., Pajor E.A. (2022). Impact of digital dermatitis on locomotion and gait traits of beef cattle. J. Anim. Sci..

[29] Woolums A.R. (2015). Feedlot acute interstitial pneumonia. Vet. Clin. N. Am. Food. Anim. Pract..

[30] Vázquez-Martínez E.R., García-Gómez E., Camacho-Arroyo I., González-Pedrajo B. (2018). Sexual dimorphism in bacterial infections. Biol. Sex. Differ..

[31] Woolums A.R., McAllister T.A., Loneragan G.H., Gould D.H. (2001). Etiology of acute interstitial pneumonia in feedlot cattle: Noninfectious causes. Compend. Contin. Educ. Vet..

[32] Hodgins D.C., Conlon J.A., Shewen P.E., Brogden K.A., Gruthmiller J.M. (2002). Respiratory Viruses and Bacteria in Cattle.

[33] Babcock A.H., Renter D.G., White B.J., Dubnicka S.R., Scott H.M. (2010). Temporal distribution of respiratory disease events within cohorts of feedlot cattle and association with cattle health and performance indices. Prev. Vet. Med..

[34] Galyean M.L., Ponce C., Schutz J. (2011). The Future of Beef Production in North America. Anim. Front..

[35] Erickson S.E., Booker C.W., Song J., Janzen E., Jelinski M.D., Schwartzkopf-Genswein K. The Epidemiology of Hoof-Related Lameness in Western Canadian Feedlot Cattle. Master of Science, University of Saskatchewan, Saskatoon Saskatchewan, August 2023. Harvest. https://harvest.usask.ca/server/api/core/bitstreams/e0439db2-ab0d-44cc-a36a-09e70eafef15/content.

[36] Woolums A.R., Loneragan G.H., Hawkins L.L., Williams S.M. (2005). A survey of the relationship between management practices and risk of acute interstitial pneumonia at U.S. feedlots. Bov. Pract..

[37] Bell J. Identifying Infection Reservoirs of Digital Dermatitis in Dairy Cattle. Doctor of Philosophy, University of Liverpool, September 2017. ProQuest Dissertation Publishing. https://www.proquest.com/docview/2344095403?parentSessionId=9rQkVaDDx8XnCB7Nol8k7phfg2oX%2Fsx6lV8%2BroKQgAw%3D&ccounted=14739&sourcetype=Dissertations%20&%20Theses.

